# A Comparative View of the Various Alterations of Structure Observed in the Fatal Cases of Continued Fever, Which Have Occurred at St. George's Hospital, between July 1st, 1826, and July 1st, 1827

**Published:** 1827-08

**Authors:** Cornwallis Hewett

**Affiliations:** one of the Physicians to that Hospital.


### FEVER.

*A comparative View of the various Alterations of Structure observed in the fatal Cases of continued Fever, which have occurred at* St. George's Hospital, *between July* 1*st*, 1826, *and July* 1*st*, 1827. By Cornwallis Hewett, M.D. one of the Physicians to that Hospital.

In this interval numerous cases of irregular intermittent fevers were admitted, some of them complicated with internal inflammations requiring venesection; but these were all cured.

Of idiopathic fever (in the continued form) there were 190 cases, out of which 165 were cured, and 26 died. A very large proportion of the latter were not admitted until a very advanced period of fever, or until after irremediable organic injuries had been effected, as may be seen in the annexed Table, which is intended to convey a concise statement of the structural lesions observed on an examination *post mortem.*

With the view of not fatiguing the reader, I have purposely refrained from entering into any minute description of the structural lesions, or of the mode of treatment. They are, however, detailed at full in the report of the Hospital Case-books, which are always accessible to the medical students. At some future opportunity, I may very possibly request the insertion of the details of some of these cases; but at present it is my wish to be as brief as possible in my remarks.

6

TABLE *shewing the Structural Lesions in Fatal Cases of Fever.*

| NAME. | BRAIN. | LUNGS. | INTESTINES. | DATE. |
|---|---|---|---|---|
| Nathaniel Shoultie ........ | Congestion of the blood-vessels of the brain, but no effusion of serum between the membranes, or in greater than the usual quantity in the lateral ventricles. | Healthy. | Very general enlargement of the follicles in the mucous membrane of the ileum and cœcum; some few of them exhibited a speck of ulceration at their apices; there were also some small cup-like cavities, formed probably by the separation or sloughing away of the enlarged follicles; the mesenteric glands were vascular. | Attacked June 20, 1826. Admitted July 3d. Obiit early in the morning of July 4th. He was evidently sinking at the time of his admission; but the abdomen was flat, soft, and not tender on pressure. |
| Fanny Slark, æt. sixteen.... | Slight effusion between the arachnoid and pia mater, and also within the theca vertebralis. | Healthy. | One follicular ulcer, with continuous purplish hue of the mucous membrane of small intestines, its surface appeared as if powdered with flour; mesenteric glands were enlarged. | Attacked July 15th. Admitted July 27th. Obiit Aug. 3d. Purulent matter under integuments of the neck, from erysipelas, which supervened after leech-bites. |
| Mary Ann Halford, ætatis twenty-four ............ | Natural. | Aphthæ and ulceration about the soft palate, epiglottis, &c. Lungs healthy. | Enlargement of the mucous follicles, (anterior to ulceration,) bearing some faint resemblance to small-pox pustules. | Attacked July 22d. Admitted July 28th. Obiit August 3d. |
| Priscilla Smith, aged about twenty-four ........... | Some small quantity of fluid between the membranes, and about three drachms in the lateral ventricles. | Structure of the lungs healthy; puriform secretion adhering to the bronchial membrane. | Shallow ulcers of the mucous glands, and apparently cicatrised; mesenteric glands only very slightly enlarged. | Attacked July 18th; was confined to bed nine days before her admission. Admitted Aug. 2. Obiit Aug. 29, with very extensive sloughing sores of back and nates. |
| Caroline Spooner, aged about fifteen. Had never taken any purgatives, and had been treated principally with diaphoretics and chalk mixture before her admission. | Effusion of serum between the membranes, and in the lateral ventricles; brain itself was slightly vascular. | Slightly congested. | Many deep ulcers, originating in the mucous follicles of small intestines; enlarged mesenteric glands. | Time of attack unknown. Admitted, in a delirious state, August 3d. Obiit August 6th. |

| NAME. | BRAIN. | LUNGS. | INTESTINES. | DATE. |
|---|---|---|---|---|
| Mary Tevenor, ætat. twenty-five .................... | About half an ounce of serum in the brain. | Healthy. | No ulcers or enlargement of mucous follicles, but purplish discoloration, with large superficial ulcers and aphthæ of small intestines and colon; mesenteric glands enlarged and vascular. | Attacked August 21st. Admitted August 25th. Obiit August 29th. |
| Henry Long, æt. twenty-five | About one ounce of serum in the lateral ventricles, and some also within the theca vertebralis. | Healthy. | Perforating ulcers, originating in ulceration of the mucous glands in ileum, and exciting peritonitis; enlarged and vascular mesenteric glands. | Attacked August 10th. Admitted August 24th. Obiit August 29th. (Vide No. 5 of the London Med. and Phys. Journal.) |
| John Dyson ............. | Considerable effusion of clear serum between the arachnoid and pia mater; not more than ordinary quantity of it in the lateral ventricles. Substance of the brain was rather firmer than usual. | Substance of lungs was healthy; mucous membrane of larynx and trachea smeared over with a thin film of coagulable lymph: and bronchi filled with frothy mucus. One ounce of serum in pericardio. | Congestion and thickening of the mucous membrane of the ileum, cœcum, and ascending colon; but no ulceration. | Attacked about Aug. 26th. Admitted Sept. 6th. Obiit Sept. 15th. |
| Henry Glover, æt. seven.... Immediately previous to the admission of this case into the hospital, the mother and two other children had died of similar attacks of fever: their residence was in the worst part of Pimlico, in the midst of dirty drains. | Dura mater adhered very firmly (even more so than is usual in children) to the cranium. Some effusion (but not so extensive as in the case of his brother,) between the arachnoid and pia mater, and within the lateral ventricles. | Healthy. | A small quantity of clear serum within the cavity, and also a faint pinkish tint of the peritoneum. Small petechial spots in the submucous structure of the ileum, and slight thickening in the neighbourhood of the ileo-cœcal valve, from more copious ecchymosis. Over this part extended a very superficial erosion (its limits being distinguishable by a line as thin as gold-beater's skin,) of the mucous membrane. No follicular enlargements. | Attacked Sept. 25th. Admitted Oct. 4th, with petechiæ, &c. Obiit Oct. 31st. |

| NAME. | BRAIN. | LUNGS. | INTESTINES. | DATE. |
|---|---|---|---|---|
| John Glover, æt. three and a half, (brother of the preceding) .............. | Head was very large, and dura mater was very firmly adherent; enormous quantity of clear serum between the membranes and in all the cavities, even in that of the septum lucidum; and also within the theca vertebralis. The substance of the brain was firm, but not exhibiting any vascular puncta. | Healthy. | Intestines were perfectly healthy in structure, but their coats were very transparent. Liver was pale; but the gall-bladder was filled with very pale bile. | Admitted, with petechiæ, Oct. 7th. Obiit Nov. 10th. |
| John Cheverell ............ | Healthy. | Sero-puriform matter in left cavity of the thorax. | Healthy. | Ailing for two months. Admitted, in nearly a comatose state, Oct. 18th; erysipelas appeared Nov. 16th. Obiit Dec. 12th. |
| John Stamford, æt. thirty .. | About two ounces of clear serum in lateral ventricles, and some also in theca vertebralis; plexus choroides was very pallid, and substance of brain was free from vascular puncta. | Empyema in right cavity of the thorax; the two lower lobes of right lung agglutinated together by coagulable lymph, and substance of the lower lobe condensed; mucous membrane at the origin of the bronchi florid and vascular. | Healthy. | Attacked Oct. 21st. Admitted Oct. 28th. Obiit Nov. 12th. The attack of pleurisy took place when he was apparently recovering from the fever: his bed was exposed to a constant draught of cold air from the door. |
| Robert Old, æt. thirty-eight | Substance of brain highly vascular; plexus choroides remarkably turgid and purplish; not more than usual moisture in the lateral ventricles. | Aphthæ about posterior fauces and epiglottis; mucous membrane of larynx, trachea, and bronchi highly vascular, with specks of superficial sloughs; at the bifurcation of the trachea, a slough of the size of sixpence. Lungs were congested, but not condensed. | Purplish discoloration of the interior of the small intestines, arising from congestion in submucous texture; some few superficial small ulcers, but no ulceration, or even enlargement, of mucous follicles. | Date of attack could not be ascertained. Admitted Nov. 1st. Obiit November 2d, within twenty-four hours of admission. |

| NAME. | BRAIN. | LUNGS. | INTESTINES. | DATE. |
|---|---|---|---|---|
| Jane Williams, æt. twenty-six. Was in perfect health until the moment of this attack of fever. | Healthy. | Healthy. | Copious deposition of coagulable lymph along the convolutions of intestines, from peritonitis, excited by an ulcer perforating the ileum, within about two inches of the ileo-cœcal valve. Numerous other ulcers, with enlarged mucous follicles, extending for a foot and a half from the ileo-cœcal valve upwards in the ileum, and about three inches in the ascending colon. Mucous membrane elsewhere was perfectly healthy. Mesenteric glands were enlarged and highly vascular. | Attacked Nov. 1st. Admitted Nov. 8th. Obiit Nov. 10th. |
| James Potter, æt. thirty-eight | Healthy. | Aphthous ulceration of soft palate, tonsils, and sides of epiglottis. Between the base of the latter and the sacculi laryngis, was a slough of some size, just on the point of its detachment. Mucous membrane below the larynx retained its natural colour and appearance, until the bifurcation of the trachea, where it became highly vascular and red, and continued so in the bronchi. The substance of the lungs was healthy. | Patches of ecchymosis in cellular membrane, uniting the mucous to the muscular coat of small intestines. Ulcers occupying the Peyerian glands, penetrating in two places to the peritoneum, but not exhibiting raised edges: these clusters of glands were scarcely more developed than natural, but the mesenteric glands were enlarged and highly vascular. | Ailing for six weeks. Attacked Oct. 28th. Admitted Nov. 11th. Obiit Nov. 23d. |

| NAME. | BRAIN. | LUNGS. | INTESTINES. | DATE. |
|---|---|---|---|---|
| *Thomas Marsden, æt. twenty-one* | Serum abundantly, and coagulable lymph partially, effused between the arachnoid and pia mater, over the convexities of both hemispheres, and around the optic nerves, and over the pons Varolii; only the usual quantity of fluid in the lateral ventricles; the plexus choroides was very turgid, and of a dusky brick-red colour. The substance of the brain was highly vascular. | Healthy. | Follicular enlargements and ulcers, with a purplish and congested condition of the submucous structure of the small intestines. The mesenteric glands were considerably enlarged, purplish, and spongy. Some few patches, of the size of a shilling, of ecchymosed blood between the laminæ of the mesentery, and a large quantity of grumous blood in the veins. | Attacked Nov. 5th.<br>Admitted Nov. 15th.<br>Obiit Nov. 20th.<br><br>He had taken no medicines until Nov. 13th. |
| Anne Milson | Large quantity of serum in the lateral ventricles; coagulable lymph about the base of the brain and pons Varolii. | Natural. | Natural. | Admitted Feb. 14th, 1827.<br>Obiit Feb. 19th. |
| Matthew Bradford | Several ounces of serum in the lateral ventricles. | Natural. | Natural. | Admitted, Feb. 14th.<br>Obiit Feb. 15th. |
| William Sawyer, æt. fifteen | Serum effused between the arachnoid and pia mater, and also in the lateral ventricles; coagulable lymph about the base of the brain, optic nerves, and pons Varolii. | Healthy. | Healthy. | Attacked with fever about Feb. 7th.<br>Admitted comatose Feb. 21st.<br>Obiit Feb. 22d. |
| Samuel North | Several ounces of serum effused between the membranes, and also copiously in the lateral ventricles. | Natural. | Natural. | Admitted April 2d.<br>Obiit April 15th. |

| NAME. | BRAIN. | LUNGS. | INTESTINES. | DATE. |
|---|---|---|---|---|
| Samuel Mills ............ | About half an ounce of clear serum in the lateral ventricles, and also some serum within the theca vertebralis. The substance of the brain was healthy. | Natural. | Large patches of ecchymosis in the cellular membrane connecting the mucous to the muscular coat of the small intestines; not the slightest abrasion of the mucous surface, nor enlargement of the mucous follicles; the mesenteric glands were highly vascular and purplish, but retained their natural size. Extensive ecchymosis in cellular membrane attaching the peritoneum to the abdominal muscles, and also between these and the external integuments: the peritoneum, however, when stripped off and examined, retained its natural appearance and transparency. | Attacked a month ago. Admitted May 8th, with petechiæ. Obiit May 26th. |
| William Brunn, æt. fifty-one | Natural. | Free from tubercles. The whole right lung was condensed, though easily lacerable: it exhibited here and there small cavities, as large as almonds, and containing a small quantity of sanious fluid, which was not retained by any well-defined cyst, but seemed to diffuse itself in the neighbouring parts. | Natural. | Attacked with fever supervening on an old cough. Admitted May 16th. Obiit May 22d. |

| NAME. | BRAIN. | LUNGS. | INTESTINES. | DATE. |
|---|---|---|---|---|
| Elizabeth Terry .......... Vide the details of this case below. | Natural. | Lungs crepitated, but exhibited occasional spots of extravasated blood under the pleura, and into the substance of the lungs. | Follicular ulceration and ecchymosis in the submucous texture of the ileum and colon. | Attacked about March 18. Admitted May 23d. Obiit June 1st. |
| Stephen Smith ............ This case is more fully detailed below. | Effusion of serum between the membranes, and within the lateral ventricles and theca vertebralis. | Substance of the lungs healthy, but the mucous membrane of the trachea and bronchi was slightly florid. | Follicular ulcers, &c. | Attacked about May 9th. Admitted May 23d. Obiit June 14th. |
| John Burt................ | Not examined. | Hepatisation. | Sero-puriform matter and coagulable lymph effused in the cavity of the abdomen, the results of peritonitis. The mucous membrane was not examined, in consequence of the haste with which the body was taken away. | Admitted May 30th. Obiit June 29th. |
| Eliza Wilson, æt. nineteen •• | Very copious effusion of serous and puriform fluid between the arachnoid and pia mater, extending over the convexities of the hemispheres and the base of the brain and pons Varolii. Scarcely more than usual quantity of fluid in the lateral ventricles. When transverse sections were made, the medullary portion was spotted with drops of blood. | The larynx and trachea were smeared over with a thin film of coagulable lymph, sufficiently thick, however, and consistent, to bear being reflected back, after having been cut through with the scalpel The subjacent mucous membrane, when thus exposed, was found highly vascular, but free from ulceration. The lungs were congested. | In the ileum, and near the ileo-cœcal valve, there were small ulcers, (but without raised edges,) in the midst of different patches of Peyer's glands, which, however, were only very slightly enlarged. There was no increased vascularity of the contiguous mucous membrane; the corresponding mesenteric glands were scarcely larger than their natural size, but were claret-coloured from increased vascularity. | Attacked five weeks ago. Admitted, in a state of coma, June 24th. Obiit June 26th. |

That these follicular ulcers of the small intestines (seen in the above report to have so often occurred,) arise spontaneously during the progress of idiopathic fever, has been already sufficiently proved, and, when once established, it will be readily admitted that, though originally the effect of fever, they must (at least during their active and irritable state) reciprocally exasperate the symptoms, and become a very effective cause of the prolongation of fever; but the doctrine that their existence, when they have lost their irritability, and assumed a tranquil condition, is not incompatible with the subsidence of the fever, and even with some semblance of convalescence, may not gain so ready a belief, and may possibly require some further proof than the mere assertion of the writer.

It would be easy to accumulate cases in proof, but the following single one will probably suffice:

Elizabeth Terry, aged about eighteen, was admitted into St. George's Hospital, on Wednesday, May 23d, 1827.—The only history which could be obtained at the time of her admission was, that she was attacked with fever last Sunday six-weeks; that she has discontinued medicines for the last week, during which the bowels have been much relaxed; but that she is now no longer subject to night-delirium. At present, she acknowledges no pain, but flinches a little on pressure of the abdomen: it is slightly tympanitic; skin is moderately warm; tongue is moist, but foul; pulse is very soft and small, beating 132.

Hydrarg. cum Cretâ gr. viij; Pulv. Ipecac. comp. gr. v. M. fiat pulvis sextâ quâque horâ sumend.—Haustus Salinus sextis horis intermediis.—Light broth.

24th.—Pulse as yesterday; tongue dryish and yellowish brown. No evacuation.

Serum lactis vinosum.—Rep$^{r}$ medicamenta.

25th.—Only one evacuation, partly liquid, but containing some small lumps of fæces, dark-brown and very fetid. Abdomen is tumid; pressure there (but also if applied over the ribs,) excites uneasiness. Was delirious during the night; has *occasional rigors*; skin at present is hot and dry; pulse is exceedingly feeble and frequent; tongue is pasty and brown.

Olei Ricini ℥ij. statim, et quartis horis, donec dej. alvus; post alvum solutam adde Tr. Opii m. iij. singulo haust. et rep$^{r}$ pulvis.

26th.—Was less delirious last night, but the evacuations have passed involuntarily; the abdomen is no longer tumid or tender; has had no more rigors; skin at present is warm and dry; tongue is rough, dry, and yellow-brown; pulse is still very feeble, soft, and frequent.

Rep$^{r}$ medicamenta.

27th.—Is in a decided rigor at present (viz. eleven o'clock A.M.) It has usually been at an earlier hour that this disposition to rigor has shown itself, which used to disappear slowly, and be succeeded by a slight increase of the temperature of the skin.

Haust. Aquæ Ammoniæ Acet., Spir. Æth. Nitrici, Syrupi āā ʒj., Tr. Opii m. xij. M. fiat haustus statim sumendus et postea, accedente rigore, repetendus.—Sulphatis Quinæ gr. ij.; Acid. Sulph. dil. q. s.; Medullæ panis q. s. ut fiat pilula, in febris remissionibus, quartâ quâque horâ repetenda.—Omitt[r] alia medicamenta.—Beef-tea, &c. frequently.

29th.—Augeatur dosis Sulphatis Quinæ ad gr. iij. in sing. pil.

30th.—Doses a great deal. Has not repeated the Æther and opiate draught more than once daily, on the return of the chilliness. Abdomen is supple, and not tender; has passed some liquid and well-coloured evacuations, the last of them unconsciously; pulse is exceedingly frequent, soft, and feeble.

Rep[r] pil.—Vini rubri ℥ vj. ex Aquâ quotidie.

31st.—Was less delirious during the night; has had five or six liquid well-coloured evacuations; pulse is 160, very small and feeble. This morning some petechiæ have appeared on the right forearm and on the surface of the tongue.

Rep[r] pil. Sulph. Quinæ tertiâ quâque horâ superbibendo haustum sequentem.—Aquæ Pimentæ ʒx.; Acid. Sulph. dil., Tr. Opii āā m. iv; Syrupi ʒij. M.—Let strong broth and wine be repeated frequently.

June 1st.—Petechiæ have also affected the left arm; blood is exuding from the gums, and the lips are incrusted with a black fur; the substance of the tongue is quite pallid and exsanguine, and its surface is streaked with a pasty brown fur; has had no stool for the last sixteen hours, and only one since the last visit. Says she has no pain whatever, and she appears at present to be perfectly clear in her intellects. The abdomen is still supple, but is again slightly tender.

Rep[r] omnia.

About eight o'clock P.M. she vomited up some black blood, to the extent of about twelve ounces, and gradually sunk.

*Sectio cadaveris*, June 2d.—The brain was healthy; there was scarcely more than the natural quantity of fluid in the lateral ventricles; the choroid plexus was pallid.

The lungs were perfectly healthy and crepitating, though occasionally marked with petechial patches, distinctly visible through the pleura. When the latter was cut through, and the lung was divided by the scalpel, it was obvious that the serous membrane was unblemished, and that the ecchymosis had taken place immediately under it, and had extended for some depth into the substance of the lung. The mucous membrane of the trachea and bronchi retained its natural colour and condition.

There were several small cup-like cavities (as if the glandulæ solitariæ had been eroded) in the mucous membrane of the colon, and also similar ulcers in the midst of some of the slightly-elevated clusters of Peyer's glands in the ileum. A purplish discoloration extended from the ileo-cœcal valve, but with a gradually fainter tint, up the small intestines, arising from the vascular congestion of the cellular membrane uniting the mucous to the muscular coat: their adherence to each other was not impaired; and the internal membrane of the upper portion of the jejunum, and the duodenum and stomach, exhibited its natural appearance. In the cavity of the intestines there was a quantity of dark blood.

The mesenteric glands were vascular, but only very little enlarged.

The other abdominal viscera were healthy.

Since making the above report, I have heard from the physician who had conducted the treatment of Elizabeth Terry's case, that, for the fortnight previous to her admission into St. George's Hospital, he had considered her so far advanced in her convalescence as not to require his further attendance.

Stephen Smith, aged thirty-three, was admitted into the hospital on Wednesday, May 23, 1827.—The history of the case was very imperfect: it was merely stated that a fortnight ago he was attacked with fever, and that for the last five or six nights he had been light-headed. The present symptoms are a loaded tongue; skin profusely moist and rather cool; frequent, soft, and small pulse; abdomen puffy, but not in the least tender on pressure. He is slow in answering, but says he has no pain any where.

Hydrargyri Submuriatis, Pulv. Cinnamomi comp. āā gr. iij.; Rhei Rad. gr. xij. M. fiat pulvis statim sumendus.—Acidi Sulph. dil. m. xij.; Syrupi Aurantii ʒij.; Aq. Cinnamomi, Aq. distillatæ āā ℥v. M. fiat haustus quartâ quâque horâ sumendus.—Diæta febrilis.

28th.—The bowels have been opened by the repetition of the powder yesterday; pulse is small, soft, and frequent.

Adde Tr. Opii m. ij. sing. haust.

30th.—Is perspiring profusely; pulse ninety-six, very soft; tongue is moist, but of a pale yellow hue. Two evacuations. No pain any where. The fever seems to have assumed a remittent type.

Sulphatis Quinæ gr. ij. in formâ pilulæ.—Superbibendo haustum supra præscriptum quartis horis.

June 4th.—He says the bowels have continued acting three or four times daily. At present (twelve o'clock) the skin is chilled, and the fingers appear benumbed, and there seems to have been, for the last three or four days, a daily return (about ten o'clock A.M.) of an imperfect kind of rigor, which has been followed by a slight increase of heat, and then by profuse perspiration. Pulse is 120, very small and feeble.

Augeatur dosis Sulph. Quinæ ad gr. iij. in sing. pil.; et adde Tr. Opii m. iv. sing. haust.

June 6th.—Good beaf-tea, &c.—Vin. rubri ℥ iij. ex Aq. quotidie.

9th.—Omitt^r pilulæ, et solvantur Sulphatis Quinæ gr. iij. in haust. præscripto, quartis horis repetendo.—Haust. Aq. Ammoniæ Acet.; Spir. Æth. Nitr. ʒj.; Tr. Opii m. xv. M. fiat haustus cras mane, accedente rigore sumendus.

11th.—Says he has had two evacuations in the last twenty-four hours, but, on examination it was found that only urine, and no feces, had been passed; abdomen is large, but soft, and not the least tender; there is some subsultus tendinum; tongue is dry and black; pulse is very frequent, but he still maintains that he has no pain. The pupils of the eyes have their natural size and contractility. Earlier in the day, he was observed to be in a profuse perspiration.

Vini rubri ℥ iij. quartis horis.—Rep^r^ medicamenta.

12th.—Two evacuations, but thrown away. No rigor to-day; but there is increase of subsultus.

Rep^r^ omnia.

13th.—Was this morning found by the nurse with spasmodic rigidity of the muscles of the neck, throat, and some parts of the body. Passed two well-coloured evacuations. At present (twelve o'clock) is perspiring profusely; the pulse is very frequent and feeble; he is unable to answer questions, and is evidently sinking.

Obiit about four o'clock.

*Sectio cadaveris,* June 14th.—A considerable quantity of clear serum had been effused between the arachnoid and pia mater, over the whole surface of the convexities of both hemispheres of the brain, and also within the lateral ventricles, and within the theca vertebralis. The plexus choroides was pallid, but the substance of the brain, when cut transversely, exhibited numerous drops of blood.

The mucous membrane of the trachea and bronchi displayed a slight increase of vascularity; the lungs were healthy and crepitating.

There were several follicular ulcers eroding the mucous membrane of the ileum and the neighbourhood of the ileo-cœcal valve; whole clusters of Peyer's glands being thickened and elevated, so as to present a raised and granulated surface, except where eroded by ulceration: the erosion seemed in this, as in every other case of follicular ulceration, to commence in the interior, and never at the margin of the elevated patches. There was no increased vascularity near these ulcers; and the mesenteric glands were of their natural size and colour.

The liver was large, but not unhealthy in its structure; the cœcum and colon were much distended with wind.

The obvious inference to be drawn from the case of Elizabeth Terry, is the possibility of the existence of these follicular ulcers during an appearance of convalescence, and that in this, as well as in numberless other instances, probably, they have been the cause, aided by some accidental indiscretion, whether in diet or in any other way, of the relapse.

The case of Stephen Smith has been related to show that, though, like the former, it commenced as a continued fever, it afterwards assumed the remittent character, and exhibited the same structural lesions of the intestines.

The subsidence of the fever in Terry was probably simultaneous with the transition of the ulcers from an irritable to a tranquil condition; and its reappearance in the remittent form seems to have arisen from some accidental renewal of irritation in them.

The conversion of the continued into the remittent form of fever in Smith, probably accompanied some similar, though less perfect, change in the condition of the ulcers into a state of tranquillity.

In these and many similar cases, the original fever seems either to subside entirely, or to assume a new character by becoming merely a secondary fever, symptomatic of the local irritation from the ulcers in the intestines.

The hectic of phthisis and the febris infantum remittens afford analogous instances of remittent fevers originating in local irritation.

It is not by any means meant to be implied that the conversion of a continued into a remittent fever is necessarily connected with this kind of ulceration in the bowels: such conversion is, on the contrary, generally and justly considered as a very favourable omen; but then the progressive amendment is sufficiently evident, which is far otherwise with cases such as have been related above.

It may be said that the same cause, which has produced the general prevalence of late of intermittents may have, in some degree, modified the symptoms, and have made the above cases assume the remittent form of fever; but I have witnessed frequent examples of the same conversion in combination with the same ulcerated condition of the bowels, before the late great increase of intermittent fevers.

It is to be remembered, too, that the formation of abscesses or internal suppurations, during or after fever, will often produce a train of symptoms not very dissimilar to those described above: care, therefore, must be taken not to confuse them.

In the two cases above related, there was no direct evidence, at the time of their admission, of structural injury having been effected in the intestines; but the history of the progress of the fever, and the experience of the coincidence of ulcerated bowels with similar symptoms in other cases of fever, induced me, from an early period, to express my suspicions that irreparable ulcerations had already taken place in the bowels.

In the case of Henry Long, the giving way of the intestine was distinctly ascertained to have taken place while he was straining on the close-stool, at four o'clock P.M. on Monday, August 28th, 1826: he had previously experienced no pain or tenderness on forcible pressure (daily applied) of the abdomen; but he was then suddenly attacked with the most acute pain and tenderness in the regions of the right ileum and hypogastrium, and he expired between three and four o'clock P.M. on the following day: the interval, therefore, between the perforation of the intestine and the death of the sufferer amounted to about twenty-three hours. The invasion of the fever took place about the 10th of August, and nineteen days before the fatal event.

A similar instance has recently occurred in my private practice, where I announced the fact of the perforation immediately after it had taken place, and ascertained the interval between it and the death of the sufferer to amount to thirty-one hours. The interval between the first commencement of the fever and its fatal termination was sixteen days.

This gentleman, aged twenty-one years, was first attacked late on the evening of the 15th of March, 1827, with fever. On the 15th, he took five grains of calomel at bed-time, and a common purgative draught on the following morning. This pill was repeated on the night of the 18th, and the purgative draught on the following morning. He also took diaphoretic antimonial draughts. On the evening of the 19th, and also of the 20th, he took a powder consisting of—

Hydrargyri cum Cretâ ℈ss.; Pulv. Ipecac. comp. gr. v.

He was first seen by me about twelve o'clock on the 21st: he did not then acknowledge any pain; his skin was harsh and dry, but not pungently hot; his pulse was 104, feeble and small; his abdomen was not the least tender, but it remained large and hard, although the bowels had been freely opened; the tongue was pasty and brown. It had been observed that since this attack he had experienced occasional rigors, which returned irregularly either towards the evening or during the night, and were followed by a slight increase of heat, with temporary delirium; and there was a permanently harsh, unperspiring condition of the skin. The following medicines were ordered—

Hydrargyri Submur. gr. ij.; Ipecacuanhæ Rad. gr. j.; Opii gr. ¼; Mucil. Acaciæ q. s. ut fiat pil. quartis horis sumenda.

Liquor. Ammoniæ Acet. ʒiij.; Tr. Opii m. viij.; Syrupi ʒj.; Aquæ distill. ℥j. M. fiat haustus, accedente rigore sumendus.

For the four following days, I was informed he appeared much better, and I did not renew my visit until the 25th, when I found no great alteration in his condition. The same treatment was continued. On the 27th, the pill was directed to be repeated every six hours, and very small doses of Castor-oil taken, if at any time the bowels were torpid in their action. On the 28th, blisters were applied behind the ears, with cold lotion to the head. On my visit at four o'clock P.M. Friday, March 30th, he stated himself to be free from all pain; but on my pressing forcibly (as I had always daily done previously without exciting any uneasiness,) on the abdomen, the sudden change of expression in his countenance bespoke the utmost agony. On inspecting the last evacuation, voided just before my visit, it was found to consist almost entirely of blood. Until this period the motions had been regularly examined, and had never excited any alarm. With the view of merely mitigating the pain, for all hope of recovery had now vanished, leeches, and then fomentations, were applied to the iliac and hypogastric regions, where the tenderness was the most acute; and these measures, assisted by opiates, obtained for him (as they had also done in the similar case of Henry Long,) a temporary relief, and he did not expire until eleven o'clock P.M. on the following day.

This gentleman had the advantage of the constant attendance of a most intelligent practitioner, in the person of his truly affectionate brother.

*Sectio cadaveris* took place April 2d.—There was a rather loaded state of the vessels of the brain, and a small quantity of fluid at its base and within the theca vertebralis; the lateral ventricles did not contain more than the usual quantity.

The mucous membrane of the trachea, and the whole of the lungs, were in a perfectly natural condition.

On opening the abdomen, some fetid gas escaped, and the descending flap of the omentum was found highly vascular, partially smeared with pus, and feebly adhering to some of the convolutions of the ileum, at spots afterwards ascertained to be places where some of the follicular ulcers had nearly perforated the peritoneum. In two or three places the perforation had been completed, and a quantity of liquid fæces was found in the cavity of the pelvis. The largest perforation was distant about four inches from the ileo-cœcal valve. There were numerous other ulcers, in various stages of their progress, extending from thence upwards about one-third of the whole length of the small intestines. None were found in the cœcum or colon. These ulcers were not surrounded by any increased vascularity, and even the valvulæ conniventes, though in parts eroded by them, retained their natural hue: for a very short distance, however, on each side of the ileo-cœcal valve, there was a purplish discoloration from the congestion in the submucous texture. The mesenteric glands were enormously enlarged (some of them being an inch and a half in length, an inch in width, and half an inch in thickness), and pulpy and purplish. There was a considerable quantity of well-coloured and nearly liquid fæces in the cavity of the intestines, and even besmearing the ulcerated surfaces.

The liver and other abdominal viscera were all healthy.

I am far from wishing to attach an undue importance to the consideration of these follicular ulcers, and am fully aware that they are the result of only one out of the many disorganising effects of fever; but while the latter, from the alarming symptoms with which they are attended, immediately command the attention of the practitioner, the former often insidiously complete the work of destruction without even exciting a suspicion of their existence. It is with the view of exposing these peculiarities in the formation, progress, and terminations of these follicular ulcers, that I have recorded the above cases and observations; and I beg to express the hope that some abler investigator may be induced to prosecute these inquiries.

